# Mechanisms of iron homeostasis in *Pseudomonas aeruginosa* and emerging therapeutics directed to disrupt this vital process

**DOI:** 10.1111/1751-7915.14241

**Published:** 2023-03-01

**Authors:** Ana Sánchez‐Jiménez, Francisco J. Marcos‐Torres, María A. Llamas

**Affiliations:** ^1^ Department of Biotechnology and Environmental Protection Estación Experimental del Zaidín‐Consejo Superior de Investigaciones Científicas Granada Spain

## Abstract

*Pseudomonas aeruginosa* is an opportunistic pathogen able to infect any human tissue. One of the reasons for its high adaptability and colonization of host tissues is its capacity of maintaining iron homeostasis through a wide array of iron acquisition and removal mechanisms. Due to their ability to cause life‐threatening acute and chronic infections, especially among cystic fibrosis and immunocompromised patients, and their propensity to acquire resistance to many antibiotics, the World Health Organization (WHO) has encouraged the scientific community to find new strategies to eradicate this pathogen. Several recent strategies to battle *P*. *aeruginosa* focus on targeting iron homeostasis mechanisms, turning its greatest advantage into an exploitable weak point. In this review, we discuss the different mechanisms used by *P*. *aeruginosa* to maintain iron homeostasis and the strategies being developed to fight this pathogen by blocking these mechanisms. Among others, the use of iron chelators and mimics, as well as disruption of siderophore production and uptake, have shown promising results in reducing viability and/or virulence of this pathogen. The so‐called ‘Trojan‐horse’ strategy taking advantage of the siderophore uptake systems is emerging as an efficient method to improve delivery of antibiotics into the bacterial cells. Moreover, siderophore transporters are considered promising targets for the developing of *P*. *aeruginosa* vaccines.

## Graphical abstract

Therapeutics directed to disrupt iron homeostasis in the human pathogen *Pseudomonas aeruginosa*.

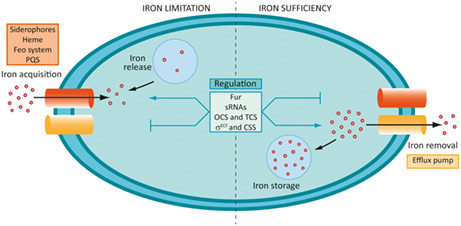

## INTRODUCTION

The pathogen *Pseudomonas aeruginosa* produces several life‐threatening infections in humans especially in immunocompromised, cancer, burn and cystic fibrosis (CF) patients, and it is a primary source of hospital‐acquired infections. The clinical relevance of this pathogen has increased over the past years due to the global rise of antibiotic resistant strains that have produced several outbreaks in hospitals (Karakonstantis et al., 2020). In fact, *P*. *aeruginosa* infections are one of the leading causes of death produced by antimicrobial resistant bacteria (Antimicrobial Resistance Collaborators, 2022). Because infections by this bacterium represent a severe threat to human health worldwide, the World Health Organization (WHO) declared *P*. *aeruginosa* a priority pathogen demanding research directed to develop new antimicrobial strategies that allow its eradication (Tacconelli et al., 2018). *P*. *aeruginosa* is metabolically very versatile and harbours multiple virulence factors that allow infection of essentially any human organ (recently reviewed in [Morin et al., 2021]). Besides the extensively studied lung infections in CF or ventilator‐associated pneumonia patients, *P*. *aeruginosa* also causes tissue infections in burns, open wounds, and post‐surgery patients; urinary tract, ear, and eye infections; and bacteraemia and sepsis. Endocarditis and meningitis produced by *P*. *aeruginosa* have been also reported. To be able to infect so many tissues and organs, this pathogen is equipped with an arsenal of virulence factors and hallmark capabilities associated with virulence (Chadha et al., 2021). Among others, the high capacity of *P*. *aeruginosa* to acquire iron is considered an important virulence determinant that facilitates its versatility and tissue tropism.

**FIGURE 1:**
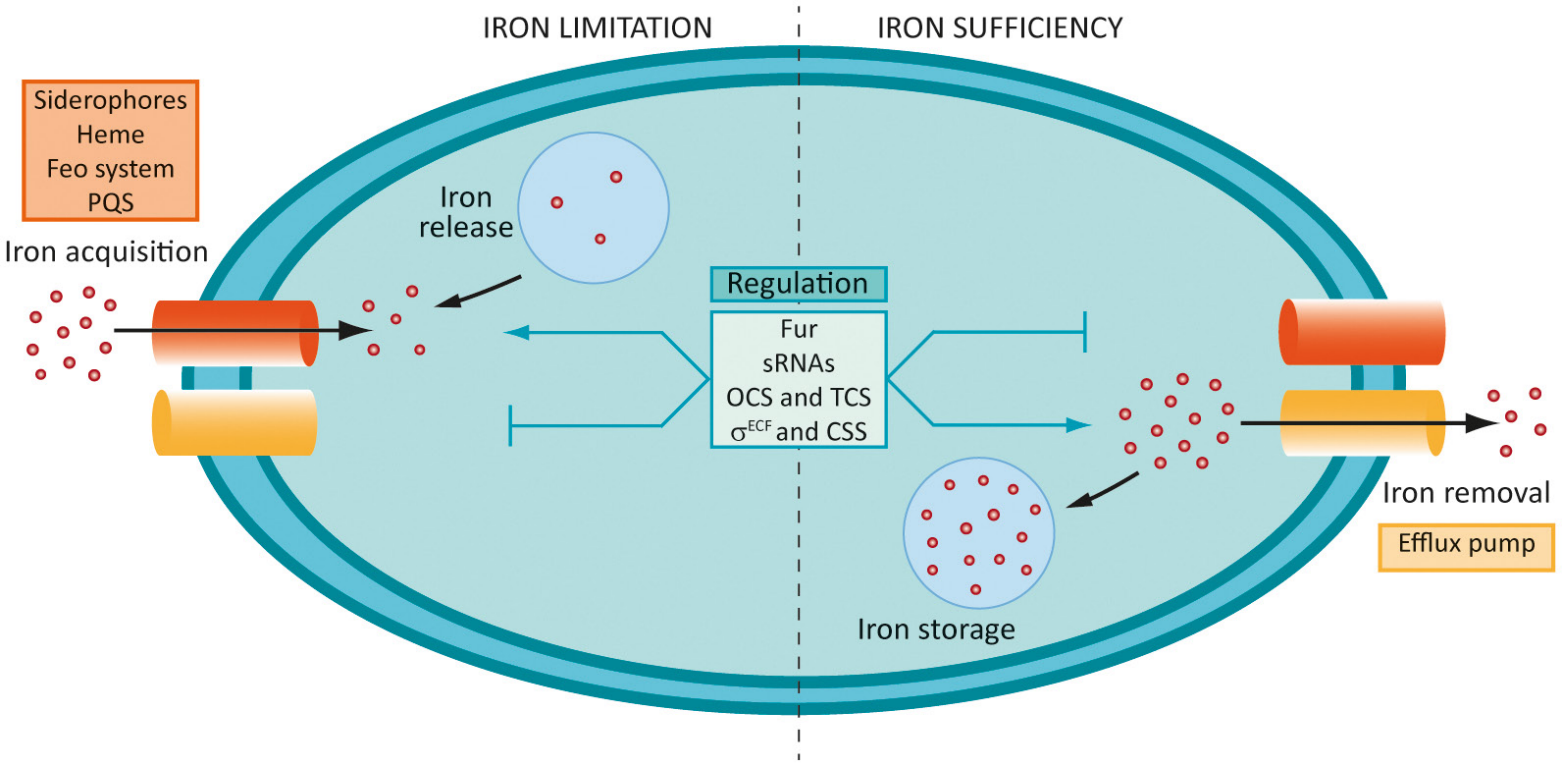
Scheme of the *Pseudomonas aeruginosa* iron homeostasis mechanisms. Under iron limitation, the production of high‐affinity iron transport systems is promoted to increase intracellular levels of iron while the iron storage and removal through efflux pump is inhibited. In excess of iron, the opposite occurs and iron storage and efflux are promoted to prevent iron overload while iron acquisition is inhibited. The regulatory systems involved in *P*. *aeruginosa* iron homeostasis are indicated. CSS, cell‐surface signalling; OCS, one‐component system; TCS, two‐component system. Adapted from (Llamas & Sánchez‐Jiménez, 2022).

Iron is an essential metal for all living organisms because it participates in several vital cellular processes and metabolic pathways as redox cofactor (Andrews et al., 2003). Iron is one of the most abundant elements on Earth. Yet despite its abundance, iron is poorly available because in the presence of oxygen, this metal oxidizes readily to its ferric (Fe^3+^) state, which at physiological pH precipitates as Fe(OH)_3_ iron hydroxide becoming insoluble. Bacteria have developed several mechanisms for iron acquisition, of which the most common strategy is the production and secretion of compounds with strong affinity for iron known as siderophores. For pathogenic bacteria like *P*. *aeruginosa*, the problem of poor iron availability increases during infection because vertebrates usually induce a ‘nutritional immunity’ response driven by metal sequestration as a reaction to the presence of invading microorganisms (Becker & Skaar, 2014). Most iron in the human body is complexed with haem in haemoproteins, especially haemoglobin, and a fraction is sequestered intracellularly in the iron‐storage protein ferritin. Extracellular iron is oxidized and transported through the body by the bloodstream complexed to the high‐affinity iron‐binding protein transferrin. The strategies used by vertebrates to limit iron availability during infection include increased production of this iron‐scavenging protein, and of haptoglobin and haemopexin that chelate haemoglobin and free haem (Cassat & Skaar, 2013). Moreover, the host also produces lipocalin, a molecule that specifically binds and neutralizes bacterial siderophores (Flo et al., 2004). Although essential for life, iron results toxic in excess due to the intrinsic ability of the reduced ferrous (Fe^2+^) form to generate free radicals through Fenton reactions, causing oxidative stress and in last instance cell death (Touati, 2000). Therefore, organisms have developed sophisticated pathways to import, chaperone, sequester, and export iron. Disruption of iron homeostasis produces either iron deficiency or iron overload, which in bacteria ultimately lead to lack of growth or even death. Because the effectiveness of a pathogen to acquire and maintain iron levels can determine the success or failure of an infection, blocking iron homeostasis is emerging as a promising strategy to prevent and/or treat bacterial infections. This review covers the mechanisms used by *P*. *aeruginosa* to maintain iron homeostasis (Figure 1) and the strategies that are currently being investigated to fight this pathogen by disrupting these processes.

## IRON ACQUISITION MECHANISMS IN *PSEUDOMONAS AERUGINOSA* AND THEIR ROLE IN VIRULENCE

### Siderophore‐mediated iron acquisition

**FIGURE 2:**
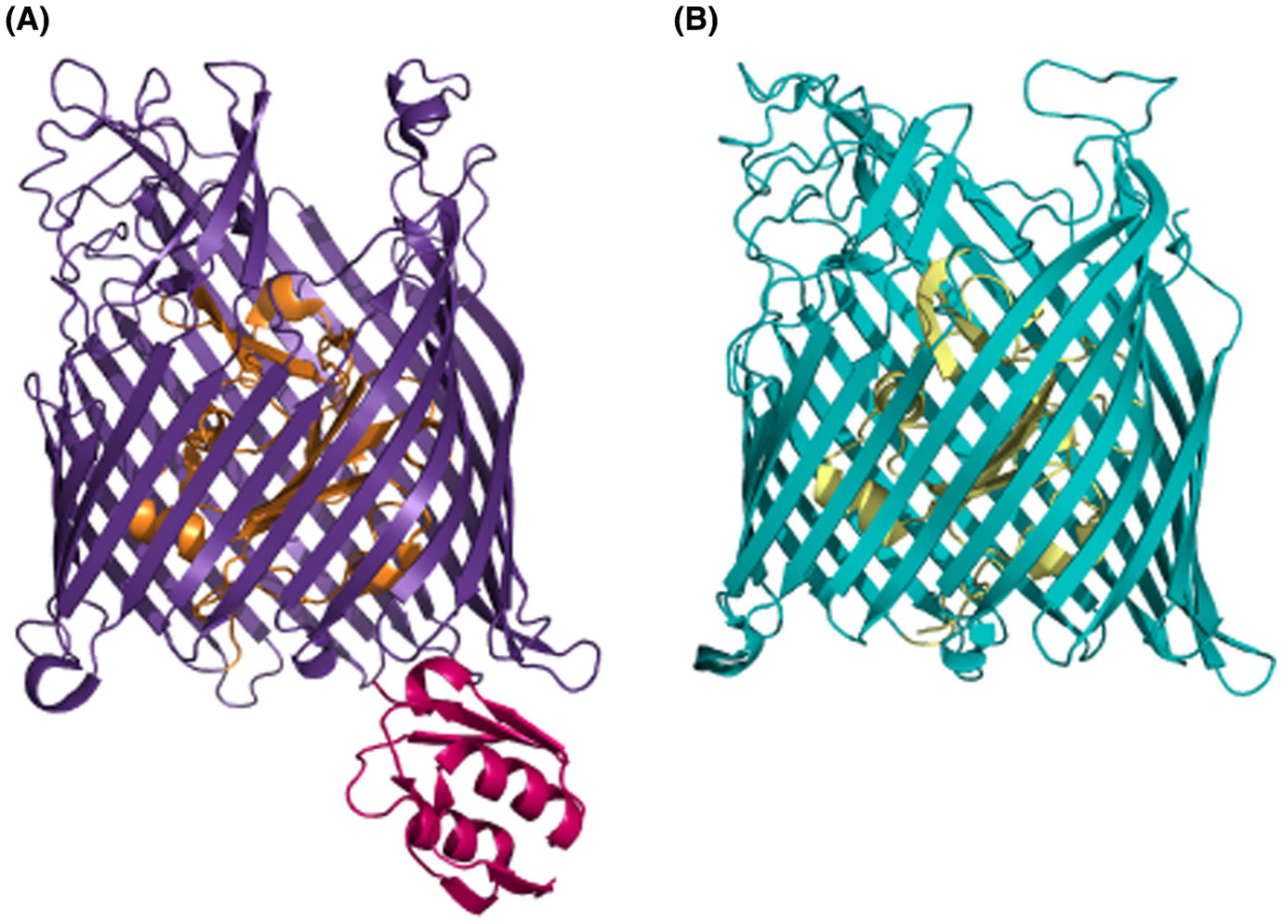
Structure of the *Pseudomonas aeruginosa* FpvA (A) and FptA (B) TBDT. TBDTs belong to the outer membrane β‐barrel protein superfamily and are composed of a C‐terminal 22‐stranded antiparallel transmembrane β‐barrel domain (in purple in FpvA and cyan in FptA) that forms a pore and an N‐terminal globular domain named plug, hatch or cork located inside the barrel that blocks the pore in the absence of the ligand (in orange and yellow, respectively). The FpvA signalling domain required for CSS in response to pyoverdine is shown in magenta. This domain is lacking in FptA which is not a CSS receptor. Structures were solved in (Cobessi et al., 2005; Wirth et al., 2007) and are downloaded from Protein Data Bank (PDB codes 2IAH and 1XKW, respectively).

Siderophores are low‐molecular weight secondary metabolites with strong affinity for iron that are produced and secreted by microorganisms in conditions of iron scarcity. In the extracellular medium, siderophores scavenge iron and form soluble Fe^3+^‐complexes that are recaptured by the bacterium via specific outer membrane receptors of the TonB‐dependent transporter (TBDT) family (Figure 2). Transport into the periplasm requires energy in the form of proton motive force (PMF) and the TonB‐ExbBD cytoplasmic membrane protein complex to transduce the energy to the outer membrane (Noinaj et al., 2010). Iron release from siderophores usually occurs via reduction to Fe^2+^. This process can take place either in the bacterial periplasm or the cytoplasm where the siderophore arrived upon transport through ABC transporters or PMF‐dependent permeases (Schalk & Guillon, 2013).

*Pseudomonas aeruginosa* produces and secretes two siderophores, pyoverdine and pyochelin, and it can utilize several xenosiderophores, that is, siderophores produced by other microorganisms, to acquire iron (Table 1). Importantly, both pyoverdine and pyochelin are key virulence factors that contribute significantly to the pathogenicity of *P*. *aeruginosa* (Takase et al., 2000). Pyochelin, which is produced at a lower metabolic cost than pyoverdine, seems to be the first siderophore synthesized by *P*. *aeruginosa* that switches to pyoverdine when the iron availability becomes more limited (Dumas et al., 2013). The Fe^3+^‐pyochelin complex does not only promote *P*. *aeruginosa* virulence through iron acquisition, but also by causing inflammation and oxidative damage in the host by its redox‐cycle activity. In fact, pyochelin is known to induce a sustained inflammatory response that produces host tissue damage during chronic *P*. *aeruginosa* infections (Lyczak et al., 2002). Pyoverdine has higher affinity for iron than pyochelin and it is thus considered the primary siderophore of *P*. *aeruginosa*. The high affinity of pyoverdine for iron allows this siderophore to steal iron even from the human iron transport protein transferrin (Wolz et al., 1994). Pyoverdine is essential for *P*. *aeruginosa* to cause acute infections (Kang et al., 2019; Meyer et al., 1996; Minandri et al., 2016; Takase et al., 2000), and, in accordance, expression of pyoverdine biosynthetic genes increases during acute infections (Damron et al., 2016; Turner et al., 2014).

**TABLE 1:** Compounds used by *Pseudomonas aeruginosa* for iron acquisition.

Iron carrier	Producer organism	TBDTs (ref)	‘Siderophore signalling’ system (ref)
*Endogenous siderophores*			
Pyochelin	*P*. *aeruginosa*	FptA (Ankenbauer, 1992; Cobessi et al., 2005)	One‐component PchR factor (Michel et al., 2005)
Pyoverdine	*P*. *aeruginosa*	FpvA and FpvB (Ghysels et al., 2004; Wirth et al., 2007)	CSS systems σ^PvdS^/FpvR/FpvA and σ^FpvI^/FpvR/FpvA (Beare et al., 2003; Lamont et al., 2002)
*Xenosiderophores*			
Aerobactin	*E*. *coli*	ChtA (IutA) (Cuív et al., 2006)	–
Cepabactin	*Burkholderia cepacia*	‐ (Mislin et al., 2006)	–
Coprogen	Fungi	‐ (Meyer, 1992)	–
Enterobactin	Enterobacteria (*E*. *coli*, *Klebsiella pneumonia*, *Shigella flexneri*, *Salmonella typhimurium*) and *Streptomyces* sp.	PfeA and PirA (Dean & Poole, 1993a; Ghysels et al., 2005; Moynie et al., 2019)	Two‐component system PfeSR (Dean et al., 1996; Dean & Poole, 1993a)
Ferrichrome	Fungi	FiuA (Llamas et al., 2006)	CSS system σ^FiuI^/FiuR/FiuA (Llamas et al., 2006)
Ferrichrysin	Fungi	‐ (Meyer, 1992)	–
Ferricrocine	Fungi	‐ (Meyer, 1992)	–
Ferrioxamine B	*Streptomyces sp*.	FoxA (Llamas et al., 2006)	CSS system σ^FoxI^/FoxR/FoxA (Llamas et al., 2006)
Mycobactin, carboxymycobactin	*Mycobacterium* sp.	FemA (Llamas et al., 2008)	CSS system σ^FemI^/FemR/FemA (Llamas et al., 2008)
Rhizobactin 1021	*Sinorhizobium meliloti*	ChtA (IutA) (Cuív et al., 2006)	–
Schizokinen	*Bacillus megaterium*	ChtA (IutA) (Cuív et al., 2006)	–
Vibriobactin	*Vibrio cholerae*	FvbA (Elias et al., 2011)	One‐component FvbR factor (Elias et al., 2011)
Others			
Ferricitrate	–	FecA (Marshall et al., 2009)	CSS system σ^FecI^/FecR/FecA (Otero‐Asman, Wettstadt, et al., 2019; Schulz et al., 2015)
Catecholamine neurotransmitters (dopamine, L‐DOPA, epinephrine, and norepinephrine)	Vertebrates	PirA and PiuA (Perraud, Kuhn, et al., 2020)	Two‐component systems PirSR (putative)
Haem	Vertebrates	PhuR, HasR and HxuA (Ochsner et al., 2000; Otero‐Asman, Garcia‐Garcia, et al., 2019)	CSS systems σ^HasI^/HasS/HasR and σ^HxuI^/HxuR/HxuA (Otero‐Asman, Garcia‐Garcia, et al., 2019)

Furthermore, *P*. *aeruginosa* is very efficient in acquiring iron from siderophores produced by microorganisms with which this bacterium shares niche and competes (Table 1). This capacity requires the production of specific TBDTs able to recognize and transport the xenosiderophore. Remarkably, *P*. *aeruginosa* produces 34 different TBDTs, most of them potentially involved in iron acquisition (Cornelis & Bodilis, 2009; Cornelis & Matthijs, 2002), denoting the potential of this bacterium to use diverse iron carriers to acquire this metal. Although still poorly investigated in vivo, acquisition of xenosiderophores is likely important during *P*. *aeruginosa* infections. Infectious diseases usually entail several microorganisms that together determine the progression of the disease. In these polymicrobial infections, microorganisms collaborate and compete modifying the growth, virulence, and/or antibiotic tolerance of the pathogens, thus affecting patient's health and the response to treatments. In the lungs of CF patients and in wound infections, *P*. *aeruginosa* is known to coexist with several bacteria and fungi, and among other mechanisms, this pathogen can inhibit the growth of these microorganisms through iron competition (Scott et al., 2019; Zhao & Yu, 2018). The ability of *P*. *aeruginosa* to use several fungal and bacterial xenosiderophores to acquire iron (Table 1) certainly enhances the competitiveness of this pathogen in these co‐infected environments.

### Acquisition of iron from the host iron carrier haem

Because most iron in mammals is complexed within the porphyrin ring of haem, the ability of acquiring iron from this host molecule is an important advantage for pathogens during infection. *P*. *aeruginosa* produces three haem acquisition systems being the only pathogen in which such a number of haem systems has been identified so far. This includes the earlier identified Phu and Has systems (Ochsner et al., 2000), and the recently characterized Hxu system (Otero‐Asman, Garcia‐Garcia, et al., 2019). The Phu and Hxu systems mediate acquisition of free haem, which is transported into *P*. *aeruginosa* cells through the PhuR and HxuA TBDTs, respectively. The Has system involves the secretion of the haemophore HasAp, which forms a haem–HasAp complex in the extracellular medium that is internalized by the HasR TBDT. During infection, haem becomes available after lysis of erythrocytes and globin degradation. *P*. *aeruginosa* secretes several factors that facilitate this process, such as phospholipase C and rhamnolipids, which have haemolysin activity, as well as proteases that can degrade haemoglobin (Ochsner et al., 2000). Although lack of the HasR receptor impairs *P*. *aeruginosa* growth in mice (Damron et al., 2016), the contribution of haem acquisition to its pathogenicity has been long considered less important than that of pyoverdine (Minandri et al., 2016). However, these conclusions predate the identification of the Hxu system and were thus based on studies performed with strains in which this haem acquisition system was intact. Importantly, absence of HasR leads to increased synthesis of the HxuA receptor suggesting that the Hxu system counteracts the lack of the Has system (Otero‐Asman, Garcia‐Garcia, et al., 2019), which may have hidden the real contribution of haem acquisition to virulence in a *hasR* mutant. In accordance with this, a recent study has shown that the Hxu system is highly expressed in *P*. *aeruginosa* clinical strains isolated from bloodstream infections, and that deletion of this system reduces the capacity of *P*. *aeruginosa* to cause sepsis while its overproduction increases it (Yang et al., 2022).

### Transport of Fe^2+^ ions via the Feo system

Both in nature and in the host, *P*. *aeruginosa* frequently inhabits environments where anaerobic niches develop, like water‐logged soils, bogs, and sediments; bottom of biofilms; or the mucus of the lungs of cystic fibrosis (CF) patients. In these hypoxic environments, the soluble Fe^2+^ ion becomes the mayor source of iron (Hunter et al., 2013). Fe^2+^ enters the periplasm of Gram‐negative bacteria likely by free diffusion through unspecific porins (Lau et al., 2016), where it must be stabilized to prevent its oxidation to Fe^3+^ and its toxicity. Although it is unclear how stabilization occurs, an interesting study showed that periplasmic cyclic glucans, a membrane‐derived oligosaccharide ubiquitously present in Gram‐negative bacteria, can bind and keep Fe^2+^ ions sequestered in the periplasm, which favours bacterial survival and growth (Javvadi et al., 2018). Transport of Fe^2+^ into the cytosol is mainly accomplished by the widely distributed Feo (from ferrous iron transport) system (Lau et al., 2016). In *P*. *aeruginosa*, this system is formed by three proteins, namely, FeoA, FeoB, and FeoC, although in most bacteria, it consists of only FeoAB and in others of FeoB alone (Lau et al., 2016). FeoB is thus the crucial component of the system. This large (~82 kDa) cytoplasmic membrane protein contains 7–12 transmembrane domains and a cytosolic N‐terminal GTPase domain that binds and hydrolyses GTP (Sestok et al., 2018). *P*. *aeruginosa* FeoB forms homotrimers and seems to contain an iron sensor domain that links GTP binding and/or hydrolysis to the opening of the pore and Fe^2+^ transport (Seyedmohammad et al., 2016).

### Acquisition of iron via the quorum sensing molecule PQS and the T6SS effector TseF

The *P*. *aeruginosa* 2‐heptyl‐3‐hydroxy‐4(1H)‐quinolone, which is known as PQS (from *Pseudomonas* quinolone signal), is a quorum sensing (QS) diffusible signal that controls gene expression in response to bacterial cell population. This signalling molecule binds to the transcriptional regulator PqsR (also called MvfR) promoting transcription of the *pqs* biosynthesis operons and that of several *P*. *aeruginosa* virulence genes (Heeb et al., 2011). Moreover, PQS contains a 3′‐OH group that enables this molecule to chelate Fe^3+^ ions at physiological pH (Bredenbruch et al., 2006; Diggle et al., 2007). Although iron chelation by PQS was originally thought to be a process for trapping extracellular iron, a mechanism for acquisition of the iron contained in PQS has been identified. This mechanism requires the *P*. *aeruginosa* type VI secretion system (T6SS) effector TseF, which seems to connect PQS with the pyochelin receptor FptA (Lin et al., 2017). Interestingly, *P*. *aeruginosa* outer membrane vesicles (OMVs) usually harbour iron‐containing PQS molecules and can therefore be used as an iron delivery vehicle, being an alternative source of iron for *P*. *aeruginosa* cells in conditions of iron starvation (Lin et al., 2017).

## MECHANISMS USED BY *PSEUDOMONAS AERUGINOSA* TO AVOID EXCESS OF IRON

### Intracellular iron storage

Given the toxicity of iron, the intracellular levels of this metal must be thoroughly controlled. The iron storage proteins ferritins are crucial in managing intracellular iron trafficking in both eukaryotic and prokaryotic cells (Honarmand Ebrahimi et al., 2015). The ferritin family is composed of the classic ferritins (Ftn), bacterioferritins (Bfr), and DNA‐binding proteins from starved cells (Dps). Ftn are present in animals, plants, fungi, archaea, and bacteria, whereas the Bfr are present only in bacteria and archaea (Andrews, 2010; Honarmand Ebrahimi et al., 2015; Lundin et al., 2012). Both Ftn and Bfr are composed of 24 subunits organized in a spherical shell that through pore and channels allows the passage of iron, chelators, oxidants and reductants, and a central cavity that can storage up to ∼3000 Fe^3+^ ions. Bfr differ from Ftn in the presence of 12 haem groups that are located at the interface of two protein subunits. Dps proteins are composed by 12 identical subunits that also form a spherical shaped structure that surrounds a hollow that can storage ~500 iron ions. *P*. *aeruginosa* harbours genes encoding a ferritin (*ftnA*, formerly *bfrA*) and a bacterioferritin (*bfrB*) (Yao et al., 2011). Earlier studies suggested that homooligomers of these two ferritins coexist in *P*. *aeruginosa* cells although the one formed by BfrB seemed to function as the main iron storage protein in this bacterium (Eshelman et al., 2017; Yao et al., 2011). However, a very recent work has demonstrated that *P*. *aeruginosa* produces a single ferritin‐like particle that is formed by heterooligomers of FtnA and BfrB (Yao et al., 2022). Interestingly, the amount of FtnA and BfrB subunits in this molecule depends on the O_2_ concentration. In aerobic conditions, the protein is mainly formed by BfrB subunits with only up to 20% of FtnA, while in O_2_ limiting conditions the proportion of FtnA subunits increases (Yao et al., 2022). Despite this variability, the final particle is consistently formed by dimers of FtnA without haem and dimers of BfrB containing a haem molecule. Mobilization of the Fe^3+^ ions stored in these molecules requires the interaction with the Bfd ferredoxin that transfers electrons to reduce the Fe^3+^ enabling the release of Fe^2+^ ions (Wang et al., 2015). Bfd binds BfrB at the interface of two BfrB subunits above a haem molecule but does not bind FtnA (Yao et al., 2012, 2022). *P*. *aeruginosa* also holds a gene encoding a Dps protein (PA0962) but this protein has not been experimentally analysed yet.

### Removal of iron through iron efflux systems

Despite being long underestimated, it is now widely accepted that active removal of cellular iron is necessary to avoid its toxicity as downregulation of iron acquisition systems and induction of iron storage systems may not always be sufficient (Bradley et al., 2020). Four iron efflux systems have been identified in bacteria to date including: (1) cytoplasmic membrane P‐type ATPases; (2) major facilitator superfamily proteins; (3) membrane‐bound ferritins; and (4) cation diffusion facilitators (CDF) (Bradley et al., 2020; Pi & Helmann, 2017). In *P*. *aeruginosa*, the only iron efflux transporter identified so far belongs to the CDF family (Salusso & Raimunda, 2017). CDF‐mediated efflux depends on the electrochemical H^+^ gradient across the cytoplasmic membrane because these proteins couple H^+^ entry with export from the cytosol to the periplasm. *P*. *aeruginosa* produces three CDF metal ion exporters, one known as AitP (from alternative iron transport protein) that exports Fe^2+^/Co^2+^ and two other that export Zn^2+^ (Salusso & Raimunda, 2017). Importantly, these transporters are needed for successful host colonization and infection (Salusso & Raimunda, 2017).

## REGULATORY AND SIGNALLING MECHANISMS CONTROLLING IRON HOMEOSTASIS IN *PSEUDOMONAS AERUGINOSA* AND THEIR ROLE IN PATHOGENICITY

### Iron sensing and regulation by fur

The ferric uptake regulator Fur is the main regulator of iron homeostasis in bacteria. This protein belongs and gives name to the FUR family of bacterial metalloregulators, which includes several other regulators that control metal homeostasis (Sevilla et al., 2021). Fur is a global transcriptional regulator that in *P*. *aeruginosa* controls either directly or indirectly the expression of hundred genes, including many iron homeostasis genes but also genes involved in virulence, respiration, central metabolism, and stress responses (Ochsner et al., 2002; Palma et al., 2003; Wilderman et al., 2004). Fur usually functions as a repressor that binds to the promotor region of target genes blocking the access of the RNA polymerase (RNAP), thereby inhibiting gene transcription. By using the Fe^2+^ ion as co‐repressor, Fur functions as an intracellular iron sensor that blocks iron acquisition and release from storages in conditions of excess of iron and promotes the reverse process when iron levels are nutritionally limiting (Figure 1). Although a conserved AT‐rich Fur binding site known as‘Fur box’ containing two overlapping inverted repeats was earlier proposed for *P*. *aeruginosa* and other bacteria (Ochsner & Vasil, 1996), recent crystal structure analyses indicate that the sequence recognized by this regulator can be highly degenerate (Deng et al., 2015). This explains why Fur can function as a global regulator and can recognize many target promoters. Besides exerting direct repression, Fur can also act indirectly by repressing the expression of positive transcriptional regulators such as σ and transcription factors (Ochsner et al., 2002; Palma et al., 2003). This allows the regulation of iron homeostasis not only in response to the intracellular amount of iron but also to other stimuli. Moreover, Fur can also function as an activator of gene expression in iron sufficient conditions. This positive control allows the Fur‐dependent expression of genes required in iron rich conditions, as for example those involved in removing excess of iron (Wilderman et al., 2004). Direct activation involves binding of Fur to the promoter region of the target gene, while indirect activation occurs via the Fur‐dependent expression of small RNAs (i.e., *P*. *aeruginosa* PrrF1 and PrrF2, see below), by the displacement of histone‐like proteins, or by blocking the binding of a second repressor (Bradley et al., 2020). Fur is an essential protein in *P*. *aeruginosa* (Visca & Imperi, 2018), which highlights the importance of controlling iron homeostasis for this pathogen.

### Regulation of iron homeostasis by small RNAs (sRNAs)

*Pseudomonas aeruginosa* codes two tandem iron‐responsive sRNAs, PrrF1, and PrrF2, which are >95% identical to each other and have redundant functions (Wilderman et al., 2004). These sRNAs are produced under iron starvation in a Fur‐dependent manner and inhibit gene translation by base pairing with the ribosome binding site (RBS) of target mRNAs. The negative regulation exerted by these sRNAs is thus responsible of the positive effect that Fur has on the expression of PrrF‐regulated genes. Targets of PrrF1 and PrrF2 include genes involved in resistance to oxidative stress (e.g., superoxide dismutase *sodB* and the catalase *katA*), iron storage (e.g., bacterioferritin *bfrB*), and intermediary metabolism (e.g., succinate dehydrogenase *sdhCDAB* cluster) (Wilderman et al., 2004). A third iron‐regulated sRNA named PrrH is encoded within the *prrF* locus (Oglesby‐Sherrouse & Vasil, 2010). Production of PrrH occurs by bypassing the PrrF1 Rho‐independent terminator and is repressed not only by iron but also by haem through the haem‐trafficking protein PhuS, which in its apo form acts as a transcriptional activator of *prrH* (Wilson et al., 2021). The PrrH regulon overlaps with that of the PrrF sRNAs (i.e., oxidative stress protection, iron storage and metabolic genes). Specific targets of PrrH include haem and pyoverdine biosynthesis genes (Oglesby‐Sherrouse & Vasil, 2010; Osborne et al., 2014). Interestingly, *prrH* and *phuS* seem to be genetically linked to, and related with virulence since both genes are found in *P*. *aeruginosa* strains but not in non‐pathogenic *Pseudomonas* (Wilson et al., 2021).

Both Fur and PrrF/PrrH sRNAs facilitate the development of *P*. *aeruginosa* infections by balancing the intracellular iron levels assuring sufficient amounts for metabolism while preventing toxicity. However, the effect of these regulators on *P*. *aeruginosa* physiology and virulence extends beyond regulation of iron homeostasis since Fur and PrrF/PrrH control many virulence determinants of *P*. *aeruginosa* (e.g., QS, oxidative stress response, pyocyanin production, motility, and biofilm formation). In fact, PrrF/PrrH sRNAs are required during *P*. *aeruginosa* infection in a murine model of acute lung infection (Reinhart et al., 2015, 2017). Moreover, these sRNAs are consistently expressed in CF lungs suggesting a role for these regulators during *P*. *aeruginosa* CF airways infection (Nguyen et al., 2014).

### Regulation of iron uptake by ‘siderophore signalling’

**FIGURE 3:**
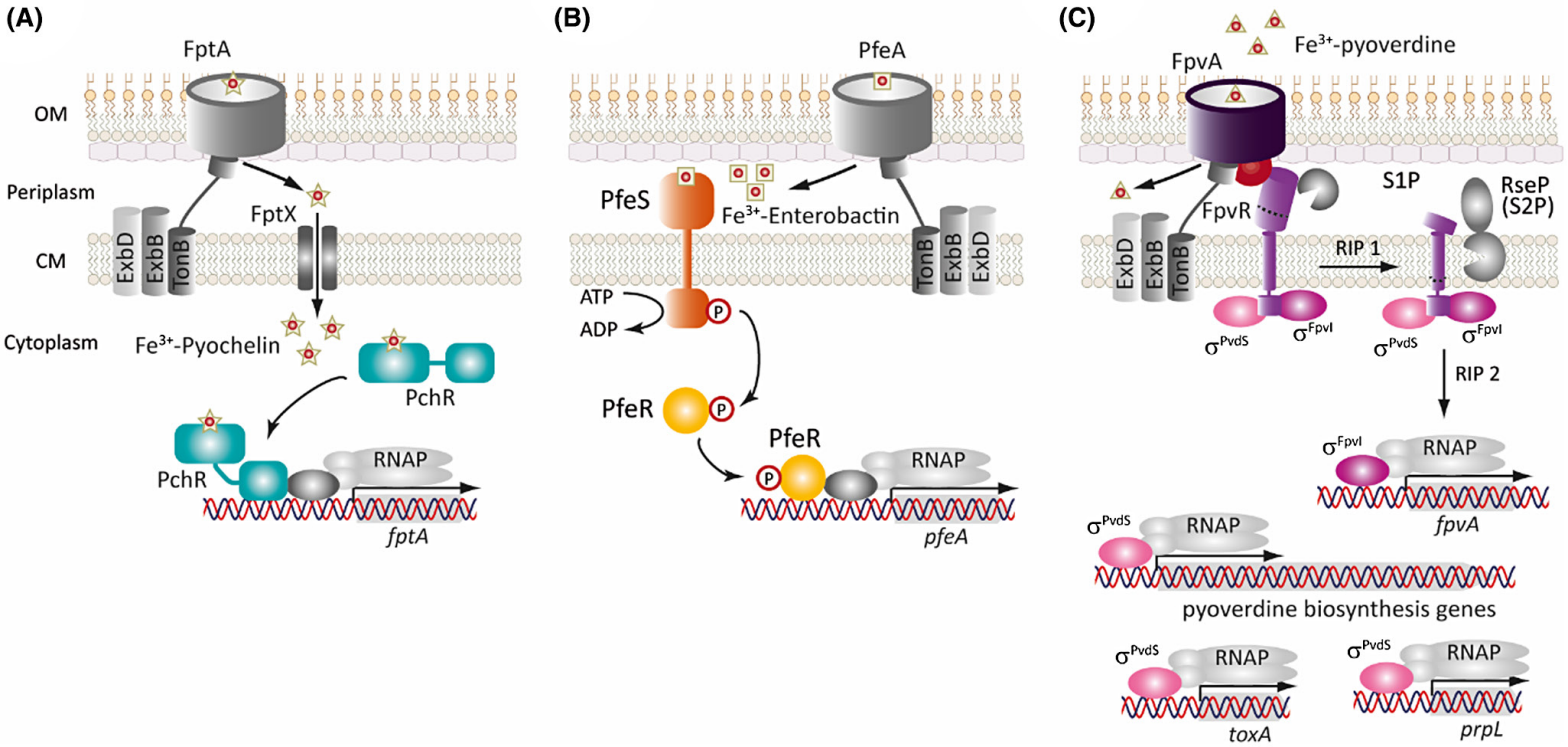
Siderophore signalling mechanisms in *Pseudomonas aeruginosa*. (A) Pyochelin‐mediated signalling via the PchR one‐component system. The Fe^3+^‐pyochelin complex is internalized into the cytosol by the transport functions of the FptA TBDT and the FptX permease. In the cytosol, the siderophore binds to the ligand‐binding domain of PchR and promotes the binding of the DNA‐binding domain of PchR to the promoter region of the *fptA* gene (among others) inducing its transcription. (B) Enterobactin‐mediated signalling via the PfeSR two‐component system. Upon internalization of Fe^3+^‐enterobactin into the periplasm via the PfeA TBDT, the siderophore binds to the PfeS histidine kinase producing its autophosphorylation at a conserved histidine residue of the transmitter domain. PfeS transfers then the phosphoryl group to a conserved aspartate residue of the PfeR response regulator. This allows the binding of PfeR to the promoter region of the *pfeA* gene (among others) inducing its transcription. (C) Pyoverdine‐mediated signalling by the Pvd cell‐surface signalling (CSS) pathway. Binding of pyoverdine to the FpvA TBDT promotes the interaction of the TonB protein with FpvA enabling the energy coupled uptake of pyoverdine, and that of the signalling domain of FpvA (red ball) with the periplasmic domain of the FpvR anti‐σ factor. This event triggers the regulated intramembrane proteolysis (RIP) of FpvR by the action of (at least) two proteases, a (still unidentified) site‐1 protease (S1P) and the site‐2 protease (S2P) RseP. This results in the release of the σ^PvdS^ and σ^FpvI^ factors into the cytosol allowing their binding to the RNAP and the transcription of target genes (see text for details). CM, cytoplasmic membrane; OM, outer membrane; RNAP, RNA polymerase.

The repression that Fur usually exerts on siderophore biosynthesis and uptake genes under iron sufficient conditions is alleviated when cytosolic iron becomes metabolically limiting. However, most of these genes are expressed only at a basal level in this condition, while maximum expression often requires the presence of the siderophore itself (Poole & McKay, 2003). This occurs by a positive regulation known as ‘siderophore signalling’ in which the siderophore or iron carrier (i.e., haem) functions as a signal molecule. *P*. *aeruginosa* uses this mechanism to control synthesis of most of its TBDTs (Llamas et al., 2006), which in this way are produced only when the cognate iron carrier is present in the environment. Synthesis of the siderophores pyoverdine and pyochelin is also promoted by siderophore signalling via positive feedback loops in which the siderophore induces its own synthesis (Lamont et al., 2002; Michel et al., 2005). This fine‐tunes the production of TBDTs and siderophores thus allowing a more precise control of iron homeostasis. In *P*. *aeruginosa*, siderophore signalling can start either at the cytoplasm, the periplasm, or the outer membrane, via one‐component, two‐component, or extracytoplasmic function sigma (σ^ECF^)/anti‐σ factor signalling systems, respectively, as exemplified below for the siderophores pyochelin, enterobactin, and pyoverdine (Figure 3).

The pyochelin‐induced siderophore signalling takes place via the activation of the PchR one‐component signalling system (Figure 3A) (Heinrichs & Poole, 1996). PchR belongs to the AraC/XylS family of transcriptional regulators, a family of cytosolic regulators that contain a non‐conserved N‐terminal domain for signal recognition and dimerization, and a highly conserved C‐terminal domain for DNA binding (Figure 3A). Internalization of the Fe^3+^‐pyochelin complex into the cytosol is required for signalling because only the iron‐loaded but not to the iron‐free form of pyochelin binds to and activates PchR (Michel et al., 2007). Upon activation, PchR binds to the DNA and promotes transcription of the pyochelin biosynthesis, transport, and regulatory (*pchR*) genes (Heinrichs & Poole, 1996; Reimmann et al., 1998; Serino et al., 1997). This increases the production of these proteins in the presence of pyochelin.

Similarly, the xenosiderophore enterobactin functions as a signalling molecule able to trigger gene expression in *P*. *aeruginosa*. Entrance of the Fe^3+^‐enterobactin complex into the periplasm activates the PfeSR two‐component signalling pathway (Dean et al., 1996; Dean & Poole, 1993b; Gasser et al., 2021). The PfeS histidine kinase perceives the presence of Fe^3+^‐enterobactin and transmits the signal to the cytosolic PfeR DNA‐binding response regulator via a phosphorelay cascade (Figure 3B). PfeR then promotes transcription of genes required for enterobactin uptake and hydrolysis (Figure 3B).

Siderophore signalling by one‐ or two‐component systems has been reported for other iron carriers (Table 1). However, the most common mechanism mediating this process in *P*. *aeruginosa* is through σ^ECF^ factors and the so‐called cell‐surface signalling (CSS) system (Table 1) (Llamas et al., 2014; Otero‐Asman, Wettstadt, et al., 2019). CSS extends from the outer membrane to the cytoplasm and involves the function of an outer membrane receptor, a cytoplasmic membrane‐anchored anti‐σ factor and a σ^ECF^ factor (Figure 3C). CSS receptors belong to a special class of TBDTs that besides the β‐barrel and plug domains contain a periplasmic N‐terminal signalling domain required for signal transduction (Figure 2A, FpvA receptor, magenta domain) (Koebnik, 2005; Noinaj et al., 2010). In the current CSS model (exemplified by the Fpv system in Figure 3C), signal recognition by the receptor (FpvA) produces the interaction of its signalling domain with the periplasmic C‐domain of the anti‐σ factor (FpvR). This triggers the proteolysis of the anti‐σ factor by a complex proteolytic cascade that involves a mechanism of regulated intramembrane proteolysis (RIP) and the RseP protease (Figure 3C) (Bastiaansen et al., 2014; Draper et al., 2011; Otero‐Asman, Wettstadt, et al., 2019). Proteolysis produces the release of the σ^ECF^ factor in the cytosol that binds to the RNAP and promotes the transcription of signal responsive genes (Figure 3C) (Llamas et al., 2014). The pyoverdine‐induced CSS system activates the function of two instead of only one σ^ECF^ factors, σ^PvdS^ and σ^FpvI^ (Figure 3C). σ^FpvI^ promotes iron acquisition by triggering expression of the *fpvA* pyoverdine receptor gene, the *hasR* haem receptor gene, and the *prrF1* sRNA that represses translation of genes involved in iron sequestration (Schulz et al., 2015). σ^PvdS^ promotes transcription of the pyoverdine biosynthetic locus and other virulence determinants including the alkaline protease PrpL and the exotoxin A (Figure 3C) (Lamont et al., 2002; Schulz et al., 2015). In accordance with its function in promoting the transcription of these important virulence factors, σ^PvdS^ is required for complete *P*. *aeruginosa* virulence (Minandri et al., 2016). As pyoverdine, extracellular haem is also used by *P*. *aeruginosa* as a signalling molecule that via the Has and Hxu CSS systems results in the activation of the σ^HasI^ and σ^HxuI^ factors (Otero‐Asman, Garcia‐Garcia, et al., 2019) (Table 1). Transcriptomic analyses recently performed in our laboratory have shown that σ^HxuI^ promotes the expression of several *P*. *aeruginosa* virulence determinants including effectors and structural components of the type II and type III secretion systems, exoenzymes and phenazine (Marcos‐Torres FJ and Llamas MA, unpublished). Thus, both pyoverdine and haem are used by *P*. *aeruginosa* not only as iron providers but also as signal molecules to trigger the production of virulence determinants, thus increasing the capacity of *P*. *aeruginosa* to cause a successful infection.

### Other regulators of iron homeostasis

There are several other regulatory systems that directly or indirectly control iron homeostasis in *P*. *aeruginosa*. This includes the product of the PA2384 gene, which is a homologue of the Fur regulator. Expression of PA2384 is induced under iron starvation and is promoted by σ^PvdS^ (Ochsner et al., 2002). Lack of PA2384 severely reduces the expression of several iron‐responsive genes (i.e., pyoverdine and pyochelin biosynthesis and transport genes, haem transport and utilization genes, iron uptake genes) while it induces the expression of genes encoding bacterioferritins, iron–sulphur‐containing proteins, and the superoxide dismutase SodB (Zheng et al., 2007). Therefore, PA2384 seems to control iron homeostasis by promoting iron acquisition and inhibiting storage, although it remains to be determined whether it functions as a direct or indirect regulator.

Several *P*. *aeruginosa* regulators that do not respond to iron but to other environmental cues also control iron homeostasis by enhancing or inhibiting transcription of the gene encoding σ^PvdS^ and thus pyoverdine biosynthesis (Llamas et al., 2014). This includes two regulators of the oxidative stress response, OxyR and the product of the PA2206 gene, which promote *pvdS* transcription (Reen et al., 2013; Wei et al., 2012). This links iron acquisition and the response to oxidative stress, which may be important to deal with excess of iron. Furthermore, the CysB transcription factor, a regulator of cysteine biosynthesis and uptake of inorganic sulphur, also functions as a positive regulator of *pvdS* transcription (Imperi et al., 2010). Co‐regulation of iron and sulphur acquisition may help to balance the intracellular levels of these two components of iron–sulphur proteins, which may be important during the biogenesis of these proteins (Imperi et al., 2010). The AmpC β‐lactamase regulator AmpR positively regulates biosynthesis and uptake of not only pyoverdine and pyochelin, but also the iron‐responsive *prrF1*/*prrH* sRNAs, the *hasAp* haemophore, and PQS biosynthesis genes (Balasubramanian et al., 2014).

The *P*. *aeruginosa* two‐component system AlgZR controls iron homeostasis by modulating the transcription of *pvdS*, *prrF2* sRNA, *hasI*, and the Fur‐like regulator PA2384 (Little et al., 2018). This regulation is influenced by the growth phase and the carbon source rather than by the iron content. Similarly, the Gac/Rsm signalling network, a multikinase regulatory cascade that favours the planktonic to sessile lifestyle transition of *P*. *aeruginosa*, control siderophore biosynthesis and uptake by modulating the expression of the *pvdS* and *pchR* regulator genes (Frangipani, Visaggio, et al., 2014; Peng et al., 2020). Activation of the Gac system favours the chronic mode of infection by allowing the translation of genes involved in chronic virulence (e.g., biofilm formation) and inhibiting that of those involved in acute infection (e.g., motility). Importantly, several studies have shown that iron supplementation enhances biofilm formation by *P*. *aeruginosa* (reviewed in [Kang & Kirienko, 2018]). Biofilm formation is a key process during *P*. *aeruginosa* infections that has important biomedical consequences since biofilms dramatically increase the resistance of pathogens to antimicrobials and the evasion of host defences (Alhede et al., 2014; Anderson & O'Toole, 2008). A pioneering study revealed that iron sequestration by the host iron carrier lactoferrin induced *P*. *aeruginosa* twitching motility and prevented biofilm formation (Singh et al., 2002). In contrast, biofilm formation by *P*. *aeruginosa* is promoted in CF patients because the most common mutation causing cystic fibrosis (the ΔF508‐CFTR mutation) leads to host cells secreting excess iron into the extracellular milieu (Moreau‐Marquis et al., 2008).

## EMERGING THERAPEUTIC STRATEGIES DIRECTED TO DISRUPT IRON HOMEOSTASIS IN *PSEUDOMONAS AERUGINOSA*

### Use of lipocalin‐based proteins to neutralize bacterial siderophores

Lipocalins (Lcn) are human glycoproteins stored in neutrophils and secreted by epithelial cells that can bind and sequester microbial siderophores. This ability makes these proteins potent bacteriostatic agents able to suppress the growth of pathogens that depend on the targeted siderophore for iron acquisition (Flo et al., 2004; Fluckinger et al., 2004; Xiao et al., 2017). Humans produce a Lcn1 known as human tear lipocalin that is secreted in tears and respiratory secretions, and a Lcn2, which is also known as siderocalin (Scn) or neutrophil gelatinase–associated lipocalin (NGAL) and is highly abundant in human serum. Lcn2 has higher affinity for siderophores than Lcn1 and it is considered an acute‐phase protein because it is highly produced during infection causing inflammation. Natural Lcn1 and Lcn2 do not recognize *P*. *aeruginosa* siderophores. However, modified Lcn2 proteins known as anticalins have been generated by reshaping the binding site to make them able to bind a wide range of targets, including *P*. *aeruginosa* siderophores (Dauner & Skerra, 2020). These engineered versions of lipocalins are currently being investigated as potential complements of antibiotic therapies.

### Utilization of iron chelators to diminish the amount of circulating iron

Another emerging therapy to treat *P*. *aeruginosa* infections is based on the use of iron chelators that sequester the free iron of the human body thus limiting the amount available for the pathogen. There are several iron chelators that are currently being investigated as potential therapeutics for their potent antimicrobial activity. One of the most promising strategies involve the use of lactoferrin, a glycoprotein of the innate immune system of mammals that is produced in human milk and several other fluids (i.e., tears, nasal exudate, saliva, bronchial mucus, gastrointestinal fluids, cervicovaginal mucus, and seminal fluid) (Zarzosa‐Moreno et al., 2020). Like Lcn2, lactoferrin is released by neutrophils at sites of infection. Lactoferrin is also recognized as an acute‐phase protein as its production increases during infections and cause inflammation. Although lactoferrin has limited effect on the growth of planktonic *P*. *aeruginosa* cells, this glycoprotein inhibits *P*. *aeruginosa* biofilm formation by restricting the amount of free iron and stimulating *P*. *aeruginosa* twitching motility (Singh et al., 2002). At present, Alaxia SAS is developing ALX‐009, a combination of lactoferrin and hypothiocyanite—a natural compound with antimicrobial activity found in saliva—to treat *P*. *aeruginosa* infections in CF patients (Reig et al., 2022; Tunney et al., 2018; Yaeger et al., 2021).

Other iron chelators with proven antimicrobial activity against different bacteria, including FDA‐approved natural and synthetic siderophores, have been evaluated to treat *P*. *aeruginosa* infections (Vinuesa & McConnell, 2021). Nevertheless, as this pathogen has an outstanding ability to use several siderophores and iron chelators as source of iron, these compounds have shown some promise in combination with antibiotics' administration rather than when used alone (Visca et al., 2013). Administration of the iron chelators desferrioxamine and deferasirox with the antibiotic tobramycin, a primary antibiotic used to treat chronic lung infections, displays a great reduction of biomass of *P*. *aeruginosa* biofilms and cell viability (Moreau‐Marquis et al., 2009). Similarly, the iron chelator N,N′‐bis (2‐hydroxybenzyl) ethylenediamine‐N,N′‐diacetic acid (HBED) enhances the activity of the antibiotic colistin against *P*. *aeruginosa* biofilms (Mettrick et al., 2020). Along the same line, the iron chelators tropolone (TRO), clioquinol (CLI), ciclopirox olamine (CO), and doxycycline (DOXY) increase the effectiveness of the peptidic antibiotic thiostrepton against *P*. *aeruginosa* and other bacterial pathogens (Chan et al., 2020). Recently, a sulfonamide that attenuates *P*. *aeruginosa* virulence thanks to its iron‐chelating activity has been identified (Yoo et al., 2022). These recent findings support that prevention of iron acquisition is a potent novel approach to treat *P*. *aeruginosa* infections.

### Use of gallium as iron mimicking

The metal gallium (Ga^3+^) has the capacity to mimick iron because it shares atomic size and oxidation state with the Fe^3+^ ion. However, it cannot be reduced and is thus unable to conduct redox reactions. By taking the place of Fe^3+^, Ga^3+^ inhibits the catalytic activity of several enzymes that require iron redox cycling, thereby blocking important metabolic processes and ultimately causing bacterial death (Chitambar, 2016; Goss et al., 2018). Gallium is also able to bind to secreted siderophores, like *P*. *aeruginosa* pyoverdine, thus decreasing the formation of Fe^3+^‐pyoverdine complexes and therefore iron acquisition (Kaneko et al., 2007). Importantly, treatment with the gallium salts gallium nitrate and gallium citrate reduces *P*. *aeruginosa* load in CF patients (Reig et al., 2022, Yaeger et al., 2021). Treatment with molecules loaded with gallium is also an interesting strategy against *P*. *aeruginosa* infections. For example, the gallium‐protoporphyrin IX (GaPPIX) compound enters *P*. *aeruginosa* cells through the PhuR haem receptor and inhibits cellular respiration by targeting haem‐dependent *b*‐type cytochromes (Hijazi et al., 2017). Similarly, loading the siderophores desferrioxamine and pyochelin with gallium inhibits the planktonic growth of *P*. *aeruginosa* better than gallium salts alone and blocks biofilm formation by reducing the amount of intracellular iron (Banin et al., 2008; Frangipani, Bonchi, et al., 2014). Because Ga^3+^ also mimicks Fe^3+^ in the human body, the therapeutic use of gallium compounds raises questions about their potential toxic effects. Gallium nitrate was the first Ga^3+^compound used as a therapeutic agent for cancer and disorders of calcium and bone metabolism, and it underwent extensive evaluation regarding its toxicity in humans. Pharmacokinetics studies in clinical trials has shown that at the actual recommended dose this compound is generally well‐tolerated by patients (Chitambar, 2010).

### The ‘trojan‐horse’ strategy: utilization of iron uptake systems to deliver antimicrobials

The so‐called ‘Trojan‐horse’ strategy exploits the need of acquiring iron to deliver antimicrobial agents inside bacterial cells (Gorska et al., 2014). This strategy is based on the conjugation of antibiotics to siderophores with the aim of improving delivery of antibiotics inside the cell by taking advantage of the pathogen's natural iron acquisition systems. This approach may be particularly suitable for *P*. *aeruginosa*, which is recognized for having an outer membrane considerably more impermeable than that of other Gram‐negative bacteria, making this pathogen intrinsically resistant to several antibiotics (Breidenstein et al., 2011). Importantly, siderophore‐antibiotic conjugates, known as sideromycins, are produced in nature by some *Actinomyces* and *Streptomyces* species (Braun et al., 2009), further supporting the potential of this strategy to kill bacteria.

Synthetic siderophore‐antibiotic conjugates have three essential components—the siderophore, the antibiotic, and the linker connecting both compounds—and all must be carefully selected to create a functional molecule. For example, most successful conjugates are based on antibiotics with periplasmic, rather than cytosolic targets, like β‐lactam antibiotics (Mislin & Schalk, 2014). This is likely due to the lower efficiency of the cytoplasmic membrane siderophore transporters to transport larger molecules, while outer membrane TBDTs are known for being able to transport big compounds. Low efficiency in the transport of the conjugate through the cytoplasmic membrane would produce its accumulation in the periplasm where the antibiotic would not have access to its cytosolic target. Regarding the siderophore, most efficient conjugates contain hydroxamate and catecholate siderophores because of their stronger capacity to induce the production of its cognate TBDT (Perraud, Cantero, et al., 2020), thus increasing the internalization of the conjugate.

Several siderophores conjugated with β‐lactams have been evaluated in preclinical and clinical studies (reviewed in Wilson et al., 2016). Of those, cefiderocol has been recently approved by the FDA and EMA for clinical use against urinary tract infections and hospital‐acquired ventilator‐associated bacterial pneumonia caused by Gram‐negative pathogens of the Enterobacteriaceae family, *P*. *aeruginosa* and *Acinetobacter baumannii* (Simner & Patel, 2020; Yaeger et al., 2021). Cefiderocol combines a synthetic β‐lactam antibiotic with an iron‐binding catechol group that improves the stability of the antibiotic and avoids its recognition by β‐lactamase enzymes (Simner & Patel, 2020; Zhanel et al., 2019). Iron‐loaded cefiderocol is internalized by *P*. *aeruginosa* cells mainly via the PiuA and PirA TBDTs (Table 1), although several other siderophore receptors can also mediate its uptake (Ito et al., 2016; Luscher et al., 2018). This supposes a great advantage because it limits the occurrence of mutations that could impair its entrance into the bacterial cell. The approval of cefiderocol for clinical use has renewed the interest in siderophore‐antibiotic conjugates. *P*. *aeruginosa* siderophores pyoverdine and pyochelin have also been evaluated for siderophore‐based conjugates (Mislin & Schalk, 2014). The most successful approaches based on pyoverdine use antibiotics with periplasmic targets because pyoverdine's fate is the periplasm where it releases the iron ion. Unfortunately, all pyochelin conjugates developed up to date are not viable due to their poor solubility (Mislin & Schalk, 2014).

### Inhibition of iron homeostasis regulatory and signalling systems

Blocking the regulatory systems controlling iron homeostasis is emerging as a promising strategy against bacterial infections. In this context, the main iron regulation systems of *P*. *aeruginosa*, that is, Fur, sRNAs, σ^ECF^ factors, and one‐ and two‐component systems (Figure 1), are interesting targets to fight this pathogen. In fact, a screening to identify compounds that inhibit siderophore production in *P*. *aeruginosa*, lead to the identification of flucytosine (5‐FC), a FDA‐approved antimycotic drug that inhibits *P*. *aeruginosa* pathogenicity by repressing transcription of the regulators PchR and σ^PvdS^ (Figure 3A,C) (Imperi et al., 2013). Comparison of the bacterial load in the lungs of 5‐FC‐treated versus placebo‐treated mice showed that 5‐FC inhibits *P*. *aeruginosa* virulence rather than bacterial viability (Imperi et al., 2013). Although this repurposed drug seemed quite promising, a more recent study showed that strains resistant to 5‐FC arise readily, which has limited the therapeutic interest in this compound (Rezzoagli et al., 2018).

Another strategy is to inhibit iron starvation σ^ECF^ factors by interfering with the components of the CSS cascade that leads to the activation of these regulators (e.g., proteases, Figure 3C). In this regard, an inhibitor of the RseP protease (Figure 3C) has been already identified in a screen using a compound collection from Merck & Co (Konovalova et al., 2018). The compound known as batimastat has been shown to inhibit the RseP‐mediated activation of the *E*. *coli* stress response σ^RpoE^ factor, leading to the accumulation of misfolded proteins in the periplasm and impaired growth (Konovalova et al., 2018). Even though the effect of batimastat on *P*. *aeruginosa* has not been assessed yet, the leading role RseP plays on CSS signalling in this bacterium (Otero‐Asman, Wettstadt, et al., 2019) makes it a promising compound.

Inhibition of haem‐mediated signalling is also a promising strategy against *P*. *aeruginosa*. Two compounds inhibiting the Has signalling system have been recently identified: an FDA‐approved oral retinoid called acitretin and a synthetic iron complex known as salophen (Centola et al., 2020; Robinson et al., 2021). Acitretin binds the Haemo oxygenase, the enzyme that catalyses the oxidative cleavage of haem generating two subproducts required for the expression of the *hasAp* haemophore gene, while salophen binds directly to HasAp. Because HasAp is the signal triggering activation of the Has signalling system and the expression of the HasR receptor, treatment with these compounds affects haem influx and signalling. The effect of these compounds in vivo has however not been evaluated yet.

### Inhibition of bacterioferritin BfrB/ferredoxin bfd union complex

A new strategy to develop novel antimicrobials against *P*. *aeruginosa* is based on the inhibition of iron mobilization from the storage protein bacterioferritin BfrB (Soldano et al., 2021). Recently, small molecules of 4‐aminoisoindoline‐1,3‐dione derivatives that inhibit the formation of the BfrB/Bfd complex have been developed. These molecules provoke cytosolic iron deficiency even in iron sufficient conditions by blocking iron release from storages. This results in inhibition of the growth of *P*. *aeruginosa* planktonic cells and killing of cells embedded in biofilms (Soldano et al., 2021). Importantly, these inhibitors enhance the effectiveness of the tobramycin and colistin antibiotics against biofilm forming cells (Soldano et al., 2021).

### TBDTs as potential targets for vaccine development

Efforts are currently being made to develop vaccines against *P*. *aeruginosa*. TBDTs have a great potential as targets for vaccine development as these proteins meet several of the desirable features including being surface exposed, regulated during infection, capable of eliciting immune responses, required for pathogenesis, and widely distributed among pathogenic strains (Wang et al., 2020). Recently, a vaccine based on peptides of the extracellular domains of the *P*. *aeruginosa* pyoverdine receptor FpvA (Figure 2A) conjugated to the immunogenic metalloprotein keyhole limpet haemocyanin (KLH) was produced and evaluated in a murine model of acute pneumonia (Sen‐Kilic et al., 2019). Nasal administration of the vaccine elicited the production of antibodies in sera and lung secretions, and increased the recruitment of dendritic and memory cells in the lungs of vaccinated mice. Importantly, vaccination considerably reduced bacterial burden in the lungs and pulmonary edema (Sen‐Kilic et al., 2019). Development of FpvA‐based vaccines could be a promising strategy to prevent *P*. *aeruginosa* infections, especially in vulnerable patients.

## CONCLUDING REMARKS

The rise of antibiotic resistance in pathogenic bacteria is one of the greatest public health problems worldwide. Although this is a natural process, the massive use of antibiotics in humans and animals has accelerated it and is the major cause of emergence of resistant bacteria in hospitals and human communities. *P*. *aeruginosa* is a nasty pathogen with a remarkable ability to infect virtually any human tissue causing infections that can be life‐threatening. Among other processes, the numerous mechanisms that *P*. *aeruginosa* has to maintain iron homeostasis (Figure 1) facilitates its versatility and allows acquisition of this essential metal in many different environments within the human body. In fact, iron acquisition is considered a main hallmark of *P*. *aeruginosa* virulence, together with biofilm formation and production of exotoxins, extracellular invasive enzymes, toxic secondary metabolites, and host colonization factors (Chadha et al., 2021). Treatment of *P*. *aeruginosa* infections can be particularly challenging because this bacterium is intrinsically resistant to multiple antibiotics and can easily acquire new resistances. The increased spread of carbapenem‐resistant *P*. *aeruginosa* strains has alerted the sanitary authorities, who have listed this species as one of the critical‐priority pathogens for the developing of new antibacterial therapies (Tacconelli et al., 2018). Novel therapeutics for the treatment of *P*. *aeruginosa* infections are based on redesigned versions of existing antibiotics, antimicrobial peptides, bacteriophages, or virulence inhibitors (Reig et al., 2022; Yaeger et al., 2021). Given the vital role of iron in *P*. *aeruginosa* virulence and survival, there are also several strategies in the pipeline for developing anti‐*P*. *aeruginosa* compounds that disrupt iron homeostasis. Most promising strategies include immune‐based therapeutics (e.g., lipocalin) and use of iron mimics and chelators (e.g., lactoferrin and gallium). Importantly, these iron‐based antibacterial strategies inhibit growth, survival, and/or virulence of *P*. *aeruginosa*, but do not kill the bacterium, thus reducing the selective pressure that leads to resistance. Moreover, iron carriers and uptake systems are being used to redesign and deliver existing antibiotics to *P*. *aeruginosa* by the so‐called ‘Trojan‐horse’ strategy. And outer membrane iron transporters (i.e., TBDTs) are used as potential targets for the development of *P*. *aeruginosa* vaccines. Despite its recognition as a clinically relevant pathogen, no vaccine against *P*. *aeruginosa* is currently in clinical development. The novel technologies successfully applied for COVID‐19 vaccine development may represent an opportunity for the generation of vaccines against *P*. *aeruginosa* and other multidrug resistant pathogens, an opportunity that will be hopefully exploited in a near future.

## AUTHOR CONTRIBUTIONS

**Ana Sánchez‐Jiménez:** Writing – original draft (equal). **Francisco J. Marcos‐Torres:** Writing – original draft (equal). **María A. Llamas:** Funding acquisition (lead); supervision (lead); writing – original draft (equal).

## CONFLICT OF INTEREST STATEMENT

The authors have no conflict of interest to declare.
